# Total aortic arch replacement with patent left internal thoracic artery graft after previous coronary artery bypass graft surgery

**DOI:** 10.1186/1749-8090-8-25

**Published:** 2013-02-18

**Authors:** Junichi Shimamura, Hidehito Endo, Hiroshi Tsuchiya, Yusuke Inaba, Yu Takahashi, Hiroshi Kubota

**Affiliations:** 1 Department of Cardiovascular Surgery, School of Medicine, Kyorin University, 6-20-2 Shinkawa, 181-8611, Mitaka, Tokyo, Japan

**Keywords:** Aortic surgery, Coronary artery bypass graft (CABG), Myocardial protection, Cardioplegia

## Abstract

A 78-year-old man, who had previously undergone coronary artery bypass graft surgery, was admitted to our department for treatment of a distal aortic arch aneurysm. A total aortic arch replacement with a patent left internal thoracic artery (LITA) graft was successfully performed without cardiac ischemic or neurological complications. Use of retrograde cardioplegia with intermittent pressure-augmented retrograde cerebral perfusion without clamping and dissecting the LITA graft were effective in myocardial and cerebral protection.

## Background

With the emergence of longer prognoses after successful coronary artery bypass graft (CABG) surgeries, reoperative and similar cases of subsequent cardiovascular surgery are becoming more frequent. These cases involve important clinical concerns including how to establish effective myocardial and cerebral protection and prevent injury to patent bypass grafts. Surgical strategies have not yet been standardized so we discuss herein a total aortic arch replacement with a patent left internal thoracic artery (LITA) graft. Retrograde cardioplegia with intermittent pressure-augmented retrograde cerebral perfusion (IPA-RCP) was used without touching and dissecting the LITA graft for myocardial and cerebral protection. The patient had a good postoperative course without cardiac ischemic or neurological complications.

## Case presentation

**Figure 1:**
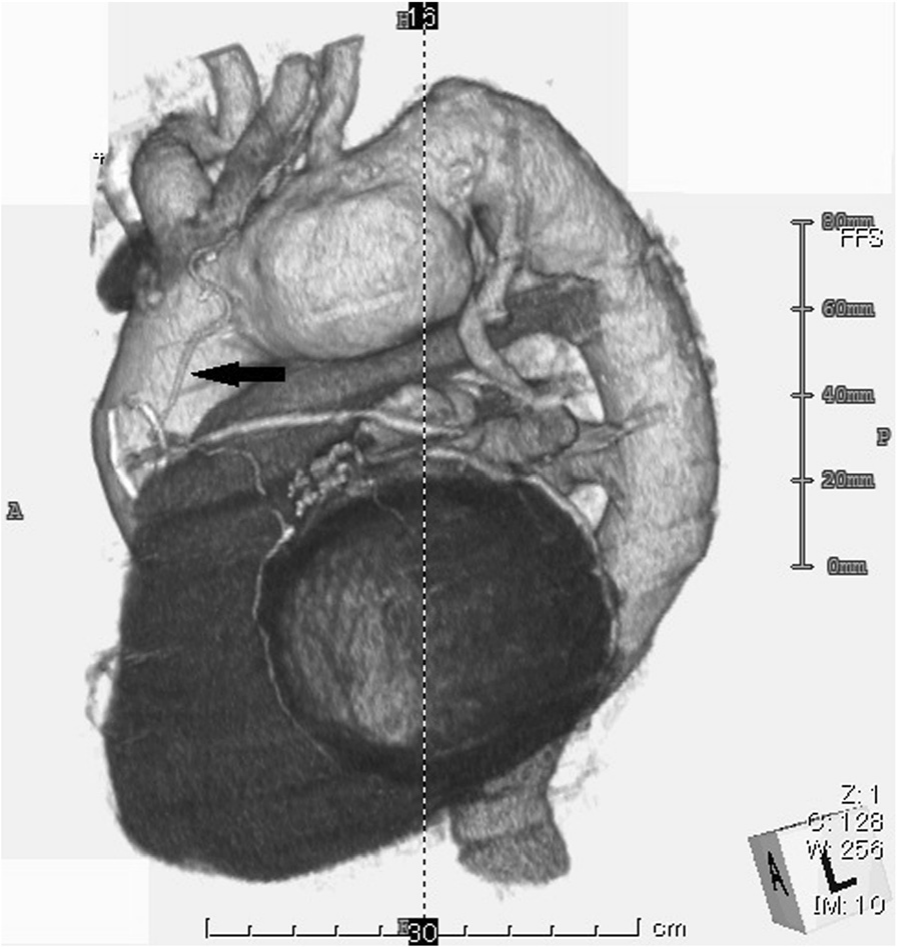
Preoperative computed tomography (CT) scan revealed saccular type aortic arch aneurysm and left internal thoracic artery bypass graft in left anterior descending artery as indicated by arrow.

A 78-year-old man, who had undergone CABG 15 years earlier, was admitted to our department for treatment of a saccular distal aortic arch aneurysm measuring 55 mm in diameter (Figure 1). A LITA graft had previously been used as a bypass in the left anterior descending artery (LAD) and a saphenous vein graft (SVG) was used sequentially in the obtuse marginal artery (OM) and posterior lateral artery (PL). Preoperative coronary angiography revealed a patent LITA-LAD with stenosis of other coronary artery segments including the SVG-OM-PL at 67%, mid right coronary artery (RCA) at 100%, proximal LAD at 100% and mid left circumflex artery at 99%. The collateral vessels were noted from left coronary artery to RCA area. The transthoracic echocardiography showed hypokinesis in the inferior area and the left ventricular ejection fraction was 58%. So our plan was to perform a total aortic arch replacement.

**Figure 2:**
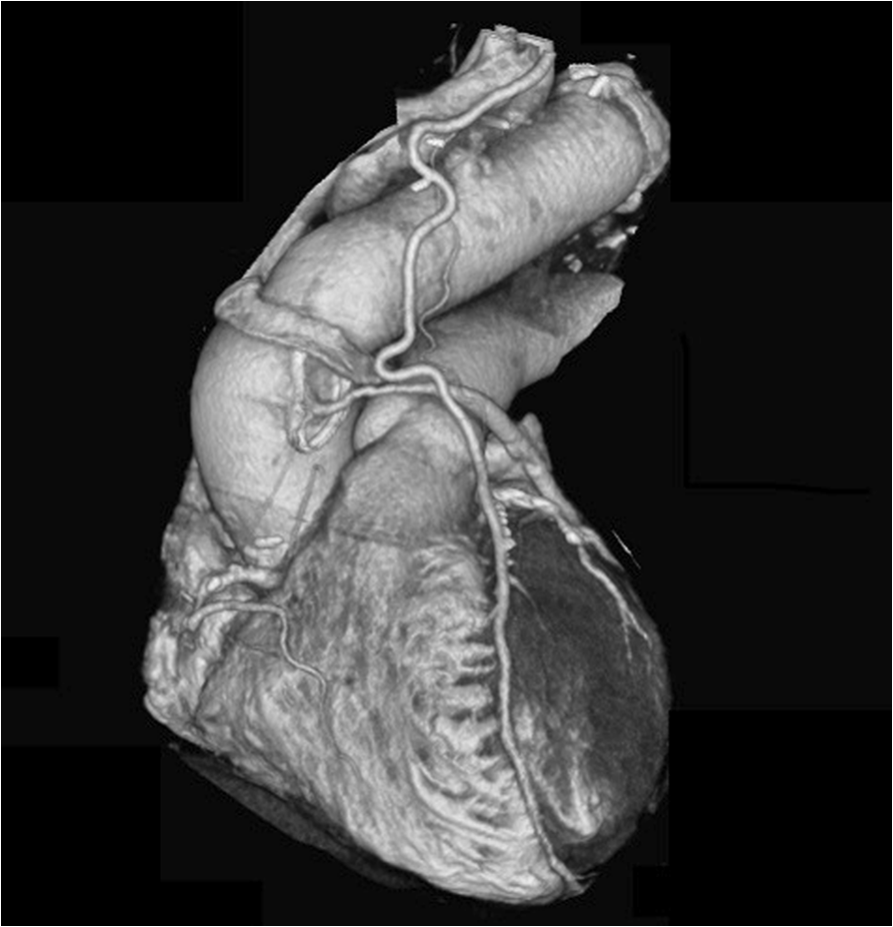
**Postoperative CT scan.** Postoperative CT scan of woven Dacron graft that replaced aortic arch.

Under general anesthesia, a repeat median sternotomy was performed with care taken not to injure the LITA graft. After systemic heparinization, cardiopulmonary bypass (CPB) was established with femoral artery cannulation and inferior and superior vena cava drainage. Left ventricular venting was also performed through the right upper pulmonary vein and 40 mEq of KCl was then injected into the cardiotomy reservoir. After confirming electrical cardiac arrest, we induced circulatory arrest. Myocardial protection was implemented by means of retrograde cardioplegia with continuous retrograde cold blood perfusion. Miotecter (Mochida Pharmaceutical Co., Tokyo, Japan) with 120 mEq/L Na, 16 mEq/L K, 32 mEq/L Mg, 2.4 mEq/L Ca, 10 mEq/L HCO3 and 160.4 mEq/L Cl was used as the cardioplegic solution. Initially, 800 ml of oxygenated blood mixed with 200 ml of Miotecter was administered and 400 ml of blood mixed with 100 ml of Miotecter was subsequently infused every 20 minutes. Continuous retrograde cold blood perfusion with oxygenated blood was performed during each cardioplegia interval by controlling perfusion pressure at <40 mmHg. IPA-RCP was initiated when the patient was cooled to 18°C. After the distal anastomosis was completed using a 26-mm four-branch woven Dacron graft (Gelweave; Vascutek-Terumo, Inchinnan, Scotland), reconstruction of the left subclavian artery (LSCA) and left common carotid artery (LCCA) were performed. Antegrade perfusion via a side branch and rewarming were started. The brachiocephalic artery was then anastomosed and perfused. Finally, the proximal anastomosis was performed (Figure 2). Weaning from CPB was uneventful. Aortic cross clamp and circulatory arrest times were 134 minutes and 79 minutes, respectively, and postoperative maximum CK-MB was 31.4 U/l. Neither postoperative ischemic nor neurological complications were noted, the patient was discharged 28 days following surgery and is currently being followed up as an outpatient.

## Comment

There have been major advances made in recent years in the surgical treatment of distal aortic arch aneurysms with substantial progress achieved in diagnosis, surgical procedures, CPB techniques and perioperative management. As a result, more severe and complicated cases are now being referred for surgery.

Subsequent cardiovascular surgery for an aortic arch aneurysm after CABG is particularly challenging because an optimal method has yet to be determined for myocardial and cerebral protection. Cases involving total aortic arch replacement with patent LITA graft after CABG have been infrequently reported because of difficulties not only in terms of the technical aspects, but also in establishing operative strategies. Injury to the LITA graft is associated with high mortality and antegrade cardioplegia increases the risk of atheromatous embolization from old grafts [1]. Using only aortic root cardioplegia is insufficient because the blood supply for the LAD area depends on the patency of the LITA graft. Previous reports have described two primary strategies [2-5] including retrograde cardioplegia with clamping and dissection of the LITA graft; and antegrade cardioplegia and hypothermic perfusion without dissection of the LITA graft.

In this case, the risk of LITA graft injury was particularly critical because the LITA graft covered most of the viable myocardium with total occlusion of the right coronary artery. Consequently, we chose RCP and retrograde cardioplegia without dissection of the LITA graft. The effectiveness and safety of retrograde cardioplegia have previously been reported by Borger et al. in reoperative cases [5]. IPA-RCP has also been demonstrated effective and facilitated a longer permissive brain ischemia time without touching the cervical branches of the aortic arch [6-9].

During circulatory arrest, cardioplegia solution flow is thought to progress through the coronary sinus, distal LAD and LITA graft to the LSCA. There will be antegrade flow in the LITA graft during the rewarming period if the pressure of the RCP is less than the pressure in the LSCA although the cardioplegia solution will continue to flow forward as during circulatory arrest if the pressure of the RCP is greater than the pressure in the LSCA.

The variable myocardium was well protected by the LITA graft because the mid RCA and proximal LAD were totally occluded and almost the entire cardioplegia solution flowed through the LITA graft. Following our protocol, the patient’s postoperative course was uneventful with no indications of myocardial damage or neurological complications. In terms of cerebral protection, there were two strategy options including the use of either RCP without LSCA perfusion as we choose or antegrade cerebral perfusion and right axillary artery perfusion with or without LCCA perfusion. Based on the results of this case, our method may be an effective strategy for successfully treating such a challenging case.

## Conclusion

Use of retrograde cardioplegia with IPA-RCP without touching and dissecting the LITA graft was effective in a total aortic arch replacement involving a patent LITA graft after a previous CABG.

## Consent

Written informed consent was obtained from the patient for publication of this case report and accompanying images.

## Abbreviations

CPB: Cardiopulmonary bypass;CABG: Coronary artery bypass graft;IPA-RCP: Intermittent pressure-augmented retrograde cerebral perfusion;LITA: Left internal thoracic artery;LAD: Left anterior descending artery;LCCA: Left common carotid artery;LSCA: Left subclavian artery;OM: Obtuse marginal artery;PL: Posterior lateral artery;SVG: Saphenous vein graft

## Competing interest

The authors declare they have no competing interests.

## Authors’ contributions

JS was the primary author of the manuscript. HK performed the operation and was a major contributor in writing the manuscript. HE, HT, YI and YT were involved in the postoperative care of the patient. All authors read and approved the final manuscript.
